# A Small Non-Coding RNA Modulates Expression of Pilus-1 Type in *Streptococcus pneumoniae*

**DOI:** 10.3390/microorganisms9091883

**Published:** 2021-09-05

**Authors:** Paloma Acebo, Cristina Herranz, Lucas Bernal Espenberger, Alicia Gómez-Sanz, María Carmen Terrón, Daniel Luque, Mónica Amblar

**Affiliations:** 1 Unidad de Patología Molecular del Neumococo, Centro Nacional de Microbiología, Instituto de Salud Carlos III, Majadahonda, 28220 Madrid, Spain; pacebo@isciii.es (P.A.); cherranz@isciii.es (C.H.); lucas.bernal.espenberger@gmail.com (L.B.E.); agsmm73@hotmail.com (A.G.-S.); 2 Unidad de Microscopia Electrónica y Confocal, Centro Nacional de Microbiología, Instituto de Salud Carlos III, Majadahonda, 28220 Madrid, Spain; mcterron@isciii.es (M.C.T.); dluque@isciii.es (D.L.)

**Keywords:** *S. pneumoniae*, Pilus-1 type, PI-1, sRNA, regulatory RNA

## Abstract

*Streptococcus pneumoniae* is a major cause of morbidity and mortality worldwide, and about 30% of the pneumococcal clinical isolates show type I pili-like structures. These long proteinaceous polymers extending from the bacterial surface are encoded by pilus islet 1 and play major roles in adhesion and host colonization. Pili expression is bistable and is controlled by the transcriptional activator RlrA. In this work, we demonstrate that the previously identified small noncoding RNA *srn135* also participates in pilus regulation. Our findings show that *srn135* is generated upon processing of the 5′-UTR region of *rrgA* messenger and its deletion prevents the synthesis of RrgA, the main pili adhesin. Moreover, overexpression of *srn135* increases the expression of all pili genes and rises the percentage of piliated bacteria within a clonal population. This regulation is mediated by the stabilization of *rlrA* mRNA since higher levels of *srn135* increase its half-life to 165%. Our findings suggest that *srn135* has a dual role in pilus expression acting both in *cis-* (on the RrgA levels) and in *trans-* (modulating the levels of RlrA) and contributes to the delicate balance between pili expressing and non-expressing bacteria.

## 1. Introduction

*Streptococcus pneumoniae* is a prominent cause of pneumonia, meningitis, septicemia, and acute otitis media worldwide [1] and is the leading cause of vaccine-preventable deaths in children under five years old (www.who.int; accessed on 2 August 2019). Nonetheless, pneumococcus is a normal component of the human commensal flora that asymptomatically colonizes the upper respiratory tracts of children and healthy adults. Human nasopharyngeal carriage is the source of transmission from person to person and serves as the first step in pathogenesis [2]. *S. pneumoniae* possesses a range of factors that facilitate nasopharyngeal colonization by enhancing host–cell attachment and have been implicated in the pathogenesis of invasive pneumococcal diseases [2,3]. These include pilus-like structures, long multimeric filaments that decorate bacterial surfaces [4,5,6,7,8].

Pili structures have been identified in a number of Gram-positive organisms. They differ from the Gram-negative pili in their genes, biochemistry, and structure as reviewed in [9,10]. Two types of pili have been identified in *S. pneumoniae*: type 1 encoded by Pilus Islet 1 (PI-1) [4,11] and type 2 encoded by Pilus Islet 2 (PI-2) [12]. Both pili are polymers of major- and minor-pilus proteins covalently linked and anchored to the cell surface [13]. Molecular epidemiology studies showed that only a small proportion of the pneumococcal population harbors pili-encoding genes in their genomes. The overall prevalence of PI-1 is 14 to 35% [8,14,15,16,17], while prevalence of PI-2 is 16–21% [12,18]. Nevertheless, several studies have suggested that pneumococcal pili provide an advantage during carriage and infection, and have a direct role in facilitating adhesion, colonization, and invasion [4,6,12,19,20,21,22]. They facilitate the formation of microcolonies and biofilms [23], stimulate the host inflammatory response during systemic infection [4], and promote phagocytosis and pneumococcal spread from local sites [20].

**Figure 1:**
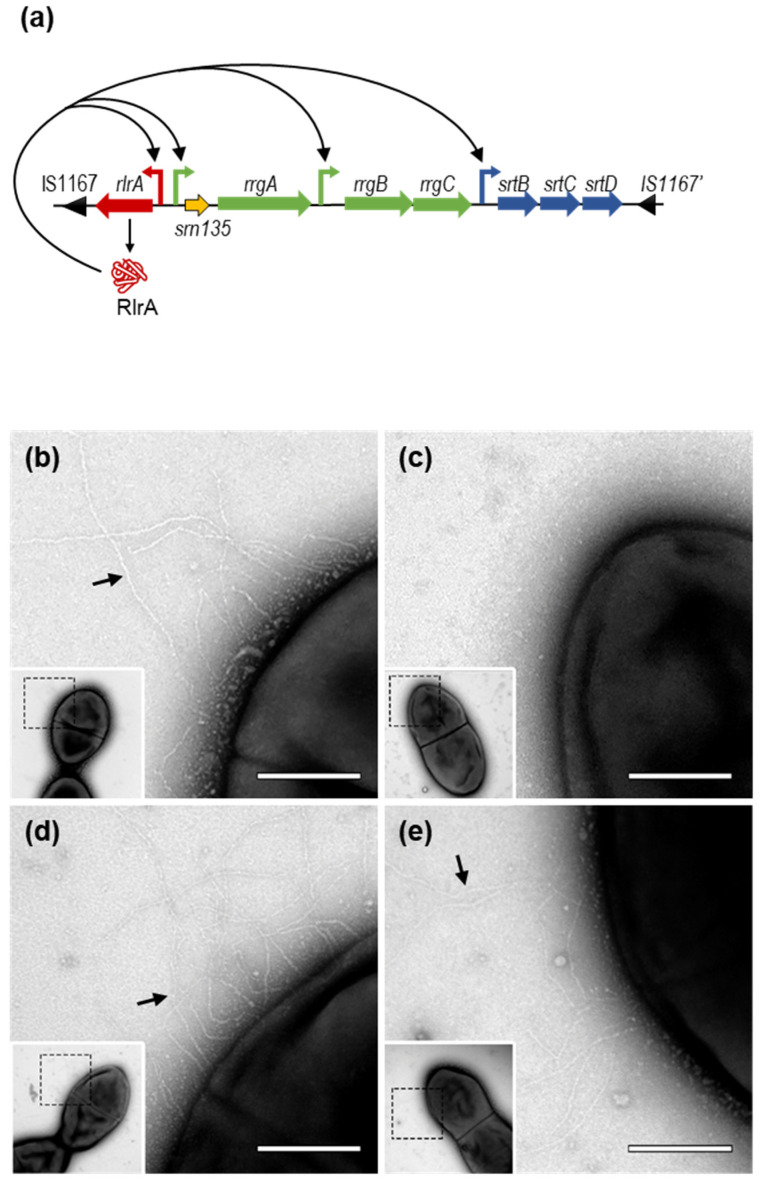
**Type-I pilus of *Streptococcus pneumoniae*.** (**a**) Schematic representation of *Streptococcus pneumoniae* TIGR4 pilus-island 1 (PI-1). Genes are represented by arrows facing the direction of transcription. The transcriptional regulator *rlrA* is red; the three structural proteins are green, and sortases are blue. The coding sequence of *srn135* is orange. The RlrA protein is represented as a red icon. Curved arrows represent the three transcriptional promoters found in the PI-1. The positive regulation exerted by RlrA as previously described is depicted by pointed arrows. Transmission electron microscopy of negative-stained strains: (**b**) TIGR4 wild type pilus-positive control, (**c**) TIGR4 Δ*rlrA* pilus-negative control, (**d**) TIGR4 Δ*rrgA*, and (**e**) the TIGR4 Δ*srn135*, showing fine pili protruding from the bacterial surface (black arrows). Bacteria were allowed to adsorb to Formvar carbon-coated grids and negatively stained with 2% (*w*/*v*) uranyl acetate. Scale bar: 250 nm.

PI-1 is a 12 kb locus that contains seven genes encoding a positive transcriptional regulator (RlrA), three pilus structural subunits (RrgA, RrgB, and RrgC), and three sortase enzymes (SrtB, SrtC, and SrtD), which covalently assemble pilus subunits on the bacterial surface [5,11,24,25,26] (Figure 1a). RrgB is the major stalk protein of the pilus and is the only protein strictly necessary for pilus formation [25,27]. RrgA, the major ancillary protein, is localized at the tip of the pilus and has been shown to be the pilus adhesin [6], whereas RrgC, the minor ancillary protein, is located at the base and serves as the pilus anchor [25,28]. RlrA is the transcriptional regulator of the pilus and activates the transcription of all seven genes in PI-1, including its own synthesis (Figure 1a), by binding to specific AT-rich sequences upstream of the four promoters of the locus [11]. In addition, other regulators have been identified, including two component systems (CbpR/S, TCS03, TCS08 and TCS09) [19,29,30,31], global regulators (MgrA, MerR, StkP and PsaR) [19,32,33], and other proteins (MntE and the Snf2 protein family member) [30,34]. The pili adhesin RrgA has also been shown to be a negative pilus regulator and this repression is likely mediated by direct interaction with RlrA [35].

Moreover, the expression of pili in pneumococci is known to be bistable, which means that within a clonal population containing the pilus-islet there is a variable proportion of pilus expressing and non-expressing cells [35,36,37]. Bimodal expression is not influenced by genotype, serotype, or growth conditions, and relays only in the levels of the RlrA. The phenotypic heterogeneity offered by bistability enables a lower energy-cost adaptation of bacterial populations to randomly fluctuating environments [38]. In fact, it has been demonstrated that the expression of pneumococcal pilus varies during colonization in mice [39], being more preferentially expressed at early colonization stages to promote adhesion and partially switched off at later stages to reduce the efficacy of the immune response. This may constitute an effective strategy during colonization/invasion and suggests that pilus expression is modulated in response to environmental cues.

Small RNA molecules with regulatory activities (sRNAs) are ubiquitous regulators that may function at transcriptional or post-transcriptional levels. They are crucial bacterial modulators of gene expression that enable the cell to adjust its physiology to environmental changes and that coordinate complex networks of stress adaptation [40,41,42]. Thus, sRNAs play key roles in the pathogenesis, colonization, and persistence of pathogenic bacteria [43]. In previous work, we identified a comprehensive list of small RNAs that are expressed in the pneumococcal clinical isolate TIGR4, 88 of which were encoded in intergenic regions of the genome [44]. Other previous and subsequent studies increased the battery of small RNAs expressed in the pneumococcus [45,46,47,48]. Inactivation of some of these sRNAs drastically affected pneumococcal virulence in a mouse model of infection, while others modulated competence or antibiotic susceptibilities [45,48,49,50,51], thus confirming that sRNAs are a major regulatory mechanism in the pneumococcus.

One of the sRNAs we identified was *srn135*, a 126 nt long RNA encoded by the positive strand of the TIGR4 genome with no homology to any family in the Rfam database [44]. Northern blot analysis confirmed its independent expression in exponentially growing pneumococci by rendering a single and specific band, and it was proposed as a novel regulatory RNA of an unknown function. Its coding sequence is located within the PI-1 in the intergenic region between genes *rlrA*, which encodes the RlrA transcriptional activator, and *rrgA*, which encodes the RrgA adhesin. This prompted us to investigate whether *srn135* participates in the regulation of pilus expression.

In this report, we demonstrate that the small RNA *srn135* corresponds to the 5′-UTR of the *rrgA* transcript and its deletion prevents RrgA synthesis. We identify several cleavage sites at the 5′-UTR of *rrgA* transcript, some of which may induce the accumulation of *srn135* as an independent RNA. Moreover, we demonstrate that higher levels of *srn135* positively regulates pilus expression, and this regulation is likely mediated by stabilization of the *rlrA* mRNA. These findings reveal that *srn135* has a dual role in pilus regulation acting in *cis*- as 5′-UTR of *rrgA* modulating translation, and in *trans*-, fine-tuning the levels of the *rlrA* transcript. Moreover, the alternative cleavage of *rrgA* 5′-UTR appears to be a key step in the balance between a productive *rrgA* messenger and the *srn135* regulator, which have opposite roles in pneumococcal pilus regulation.

## 2. Materials and Methods

### 2.1. Bacterial Strains, Plasmids, Cells Lines, and Growth Conditions

Bacterial strains and plasmids used in this study are described in Table 1. The parental strains for genetic modifications were TIGR4 and R6. Cloning experiments were carried out in the R6 strain of *S. pneumoniae*. PCR fragments were cloned into pGEM^®^-T Easy vector (Promega, Madrid, Spain) and *Escherichia coli* DH5α (F^−^, ø80d*lacZ*ΔM15, Δ(*lacZYA-argF*) U169, *deoR*, *recA*1, *endA*1, *hsdR*17(rK^−^, mK^+^), *phoA*, *supE*44, λ^−^, *thi*-1, *gyrA*96, *relA*1) was the strain selected for general cloning assays. Pneumococcal cells were grown as a static culture either in a Todd–Hewitt medium supplemented with 0.5%yeast extract (THY) or in a casein hydrolase-based medium (AGCH) supplemented with 0.3% sucrose and 0.2% yeast extract (A + SY). Human lung epithelial cells (A549) were maintained in complete RPMI, containing RPMI plus 1% Glutamine, 1% Hepes (Lonza, Basel, Switzerland), supplemented with 10% FBS (Lonza) and Penicillin–Streptomycin (Lonza). Cells were grown in a humidified incubator at 37 °C with 5% CO_2_. 

### 2.2. Transformation of Pneumococcal Cells

*S. pneumoniae* TIGR4 cells were transformed using a standard protocol based on [53,54]. Briefly, bacteria were grown in pH 6.8 THY medium to an early log phase (OD_580_ = 0.015). Cells were harvested by centrifugation (10 min, 12,000× *g*, 4 °C) and concentrated 10-fold by suspension in THY pH 8 supplemented with 1 mM CaCl_2_ and 0.2% BSA. Bacteria were treated with 200 ng/mL of CSP-2 (Competence Stimulating Peptide-2) and incubated at room temperature for 5 min. DNA was added and the culture was incubated at 37 °C for 2 h in a 5% CO_2_ atmosphere. Cultures were plated onto blood agar plates containing erythromycin 1 μg/mL or tetracycline 3 μg/mL. Transformants were grown in THY at 37 °C and 5% CO_2_. *S. pneumoniae* R6 cells were transformed as described previously [55], and transformants were plated on A + SY media plates containing 1% agar. Incubations were performed at 37 °C in a 5% CO_2_ atmosphere.

### 2.3. Construction of Strains and Plasmids

The *srn135*, *rlrA*, and *rrgA* deletion strains were constructed using the Cheshire cassette for markerless gene deletion [56]. Up- and downstream fragments for each deletion were generated by PCR using the primer pairs listed in Table 1. The fragments were cut with Mlu I and Nco I and ligated to a Cheshire fragment carrying the erythromycin resistance marker prepared with the primers 9 and 10 as described [56]. All fragments were purified, mixed together, ligated with T4 DNA ligase, and used to transform TIGR4. Subsequent treatment with 0.5% fucose to remove the resistance cassette was performed as previously described [56].

The *srn135* coding sequence was cloned into the pROM plasmid previously constructed [51] under the control of the constitutive P*_vegT_* promoter to obtain the pveg135 plasmid. For this purpose, a fragment corresponding to the first 197 nucleotides (nt) of the *rrgA* gene was amplified from the TIGR4 genome using primers 135F-blunt and 135R-Bam (Table 2). This fragment spans almost the whole 5′-UTR of *rrgA* from the +1 site (G) identified in this report to the 22 nt upstream of the ATG codon and contains the coding sequence of *srn135* as identified by massive sequencing. The resulting PCR was digested with BamH I and ligated to the pTP3 [49] vector previously linearized with Pme I (blunt end) and BamH I (cohesive end), obtaining the pTP3-135 plasmid with the *srn135* coding sequence under the control of the P*_vegT_* promoter. A fragment containing the P*_vegT_* sequence followed by *srn135* was then amplified from the pTP3-135 plasmid using pveg-Hind and 135R-Bam primers and further ligated to pROM plasmid previously digested with Hind III and BamH I enzymes. The resulting pveg135 plasmid was introduced in TIGR4 cells by transformation to obtain the TIGR4(pveg135) strain, and positive clones were confirmed by sequencing.

To construct the R6*rlrA* strain, a 1997-bp chromosomal fragment containing the entire *rlrA* gene including its natural promoter and RlrA binding sites were amplified with primers rlrA-Sph and rlrA-Bam (Table 2) and cloned into the integrative pTP2 vector [49] previously digested with Sph I and BamH I. The resulting pTP2-rlrA plasmid was introduced in the non-piliated R6 strain by transformation. Integration of pTP2-rlrA in the R6 chromosome occurred between loci *spr0564* and *bgaA*, thus obtaining the R6*rlrA* strain harboring an ectopic copy of the *rlrA* gene in the chromosome. Transformants were confirmed by DNA sequencing. The R6*rlrA*(ROM) and R6*rlrA*(pveg135) strains were constructed by transformation with pROM or pveg135 plasmids, respectively.

### 2.4. RNA Isolation and Northern Blot

Overnight cultures of *S. pneumoniae* TIGR4 cells were diluted in pre-warmed THY up to a final OD_650_ of 0.015 and incubated at 37 °C until OD_650_ ~ 0.2. At this point, 30 mL of culture was harvested by centrifugation (20 min, 8000× *g*, 4 °C). Pneumococcal cells were lysed by incubation in 500 µL lysis buffer (50 mM Tris pH 8.0, 10 mM EDTA pH 8, 0.1% sodium deoxycolate) plus 1 µL of RNase protector (Roche LifeScience, Barcelona, Spain) for 5 min at 37 °C followed by treatment with TriPure reagent (Roche LifeScience) as described by the manufacturer. Total RNA was then treated with 10 U DNaseI (Roche LifeScience) per 50 µg of RNA for 50 min at 37 °C. The reaction was stopped by the addition of 8 mM EDTA pH 8 and DNase I was heat-inactivated (10 min, 75 °C). RNA integrity was evaluated by gel electrophoresis and its concentration was determined using a Nanodrop 1000 machine (Nanodrop Technologies-ThermoFisher Scientific, Madrid, Spain).

Northern blot analysis was performed as previously described [44]. Briefly, total RNA samples (10 µg) were separated under denaturing conditions by 6% PAA/7 M urea gel in a TBE buffer. Transfer of RNA onto Hybond-N+ membrane (Amersham-Merck, Madrid, Spain) was performed by electroblotting overnight at 18 V in a TAE buffer, and RNA was UV-crosslinked immediately after transfer as described [44]. The membrane was then hybridized in PerfectHyb Plus buffer (Sigma-Merck, Madrid, Spain) for 16 h at 68 °C with biotinylated probes. Detection was carried out by autoradiography using a Phototope®-Star Detection kit from New England Biolabs (Beverly, MA, USA). Biotinylated probe synthesis and labeling were performed as previously described [44], using PCR products obtained with the primer pair listed in Table 2, as templates.

### 2.5. Quantitative Real-Time PCR

Reverse transcription reactions were performed as previously described [57] using the Transcriptor First Strand cDNA Synthesis Kit (Roche LifeScience) and 60 μM of random hexamers. A mock cDNA synthesis reaction was performed, with the reverse transcriptase replaced by water (-RT). Quantitative PCR reactions were carried out as previously described [57] using the LightCycler^®^ 480 System for Real-Time PCR (Roche LifeScience) and the primer pairs listed in Table 2. Relative changes in gene expression were calculated by the 2^−ΔΔCt^ method as previously described [58] using 16S rRNA as an internal reference gene. Two technical and at least three biological replicates were performed.

### 2.6. Rapid Amplification of cDNA Ends (RACE) Experiments

5′ RACE assays were performed essentially as previously described [59]. Total RNA extracted from TIGR4 wild type and Δ*rrgA* mutant strains was split into two halves; one was treated with Tobacco Acid Phosphatase (TAP) (Epicenter-tebu-bio, Barcelona, Spain), while the other remained untreated. Both halves were ligated to 500 pmol of RNA-adaptor (Table 2) at 17 °C for 16 h, then phenol chloroform-extracted and ethanol-precipitated. Ligated RNA was reverse transcribed with 25 pmol of gene-specific primers (RT-RACE for the wild-type strain and primer 10 for the Δ*rrgA* mutant) (Table 2), using Transcriptor Reverse Transcriptase (Roche LifeScience) for 30 min at 55 °C, followed by RNase H treatment at 85 °C for 5 min. Two microliters of RT reaction were first amplified with Fast Star Taq DNA polymerase (Roche LifeScience) and 20 pmol of each gene-specific (RT-RACE for the wild-type strain and primer10 for the Δ*rrgA* mutant) and the RACE-5′ adapter-specific primer (Table 2) in a PCR-outer reaction. These PCR products were purified using NZYGelpure (Nzytech, Lisboa, Portugal) and 4 µL were then subjected to a second amplification (PCR-inner) with internal gene-specific (wt-inner for the wild type and rrgAKO-Mlu for the Δ*rrgA* mutant) and adapter-specific (RACE-5′-inner) primers (Table 2). Products from PCR-inner were separated on 4% of MetaPhore^TM^ Agarose gels (Lonza) and bands of interest were excised, gel eluted, and either directly sequenced or cloned into pGEM^®^-T Easy for further sequencing with SP6 and T7 primers (Table 2).

### 2.7. Negative Staining and Transmission Electron Microscopy

For negative staining, bacteria were grown in THY medium until an OD_650_ of 0.3 and a 10-fold concentration in 0.9% NaCl. Small aliquots of this suspension were then adsorbed for 1 min onto glow-discharged 400 mesh carbon-coated Formvar microscopy copper grids. The bacteria were allowed to settle (1 min), negatively stained with 2% (*w*/*v*) uranyl acetate for 40 s, and air-dried. Images were recorded at nominal magnifications of 4000–75,000× on a CCD (Charged Coupled Device) ES1000W Erlangshen camera (Gatan, Pleasanton, CA, USA) on a JEM-1011 electron microscope operated at 100 kV (JEOL, Tokyo, Japan).

### 2.8. Adherence to Epithelial Cells

A549 cells grown in complete RPMI media were seeded in a 24-well polystyrene plate (Corning, Madrid, Spain) at a density of 1 × 10^5^ cells per well and cultivated in a humidified incubator at 37 °C with 5% CO_2_ to give cell monolayers of fully confluent cells (~2 × 10^5^ cells per well). Prior to infection, cells from one well were harvested with 0.25% trypsin-EDTA and counted using a Neubauer Chamber. The remaining wells were washed three times with phosphate-buffered saline (PBS), and 1 mL of antibiotic-free media was added to each well. After 30 min of incubation, the A549 cells were subjected to a multiplicity of infection (MOI) of 50 bacteria per cell with the TIGR4 wild-type strain or the Δ*rrgA* and Δ*135* mutants, previously suspended in RPMI medium. Bacteria were centrifuged onto host cells at 120× *g* for 2 min, and infection was carried out for 1 h at 37 °C and 5% CO_2_. Non-adherent bacteria were removed by rinsing the cells three times with PBS. For further enumeration of adherent bacteria, epithelial cells were subjected to lysis with 0.025% saponine/PBS. Adherent bacteria were evaluated by serial plating on blood agar plates and the percentage of adherent bacteria relative to the initial inoculum was determined. Two replicates were performed on each experiment and the final adherence values were the mean of at least three independent experiments.

### 2.9. Total Protein Extraction and Western Blotting

Bacterial cultures were grown in the same conditions as described above for RNA extraction. Four mL culture samples were collected by centrifugation. The cell pellet was suspended in 50 μL TE buffer supplemented with 0.15% sodium deoxycholate and 0.01% SDS. After 15 min incubation at 37 °C, SDS was added to a final concentration of 1%. Protein concentration was determined using Bio-Rad Protein Assay and 10 µg of protein was separated in NuPAGE^®^ Novex^®^ 4–12% Bis-Tris Protein Gels (Invitrogen-ThermoFisher Scientific, Madrid, Spain) at 200 volts (2 h for RrgB and 1 h for RrgA) following the manufacturer’s recommendations. After electrophoresis, proteins were transferred to the nitrocellulose membrane (Amersham-Merck) by electroblotting for 1 h at 100 volts. After blocking with 5% milk in TTBS, membranes were probed with a 1:50,000 or 1:200,000 dilution of guinea pig anti-RrgA or rabbit anti-RrgB (kindly provided by Prof. R. Malley from Children’s Hospital Boston), respectively. Secondary anti-guinea pig and anti-rabbit peroxidase-conjugated antibodies were used (1:10,000). Immunodetection was conducted using the Amersham ECL Prime Western Blotting Detection Reagent (Amersham-Merck). Band intensities from three independent replicates were measured to obtain a quantitative measure of pilus proteins expression.

Membrane stripping was performed with 2% SDS in 60 mM Tris HCl pH 6.8 supplemented with 100 mM β-mercaptoethanol at 50 °C for 45 min. After extensive washing with TTBS, the membrane was blocked again and probed with rabbit anti-GyrA antibody (1:5000).

### 2.10. Flow Cytometry

Flow cytometry assays were performed essentially as previously described [35]. Bacteria recovered from 1.5 mL liquid cultures grown in THY up to an OD_650_ of 0.3 were washed with PBS and fixed with 1 mL of 4% paraformaldehyde for 45 min at 4 °C. After a second wash with PBS, cells were suspended in 1.5 mL of cold GTE buffer (50 mM glucose, 1 mM EDTA, and 20 mM Tris HCl pH 7.5). The bacterial suspension was divided into two aliquots of 125 µL each and centrifuged. Pellets were blocked with 100 µL of PBS, supplemented with BSA 1% for 1 h at 4 °C, washed again with PBS and incubated with rabbit anti-RrgB (sample) or rabbit anti-IgG (isotype IgG control) diluted 1:10,000 in PBS + 1% BSA protein for 30 min at 4 °C. Bacterial suspensions were then centrifuged, washed with PBS +1% BSA, and further incubated with Alexa Fluor 488 goat anti-rabbit IgG (Molecular Probes-ThermoFisher Scientific, dilution 1:1000), as a secondary antibody. Bacterial staining was analyzed on a FACS-Calibur cytometer (Becton Dickinson, Madrid, Spain). For each sample, at least 20,000 events were recorded, and the percentage of pilus-1-positive bacteria was calculated with CellQuest Pro 2.0 software (BD Bioscience, Madrid, Spain). Sera from mice immunized with PBS plus adjuvant were used as a negative control (IgG isotype control).

### 2.11. Indirect Immunofluorescence (IIF) and Confocal Microscopy

Bacterial suspensions were fixed and prepared as described above for flow cytometry. Indirect Immunofluorescence assays were essentially performed as previously described [60]. One hundred microliters of cells suspended in 0.4 mL of GTE were deposited onto 10 mm diameter glass coverslips previously coated with 50 µg/mL polylysine for 1 h and incubated for 10 min at room temperature. Unattached cells were aspirated, and attached cells were treated with 100 μL of 0.2% Triton X-100 in PBS (PBS-T) for 10 s. After aspiration of PBS-T, the coverslips were immediately immersed in prechilled −20 °C methanol, kept at −20 °C for 10 min, and air-dried completely. Further incubations were performed at room temperature with 100 µL of each reagent as follows: 5 min with PBS-T, 1 h of blocking with PBS-T-M (5% skim milk powder (Oxoid-ThermoFisher Scientific) in PBS-T), two 10 s washes, and one 5 min wash with PBS. The coverslips were then incubated with rabbit anti-RrgB (1:40,000) and guinea pig anti-RrgA (1:5000) polyclonal antibodies diluted in PBS-M (5% skim milk powder in PBS) for at least 1 h in a humid chamber at room temperature, and washed twice with PBS for 10 s and once for 5 min. Then, the coverslips were incubated in the dark with a 1/1000 dilution of secondary Alexa Fluor 488 goat anti-rabbit IgG and Alexa Fluor 647 goat anti-guinea pig IgG (Molecular Probes-ThermoFisher Scientific) antibodies. After washing twice with PBS for 10 s and once for 5 min, they were incubated with 4′,6-diamidino-2-phenylindole (DAPI; final concentration, 2 μg/mL) for 15 min, washed twice with PBS for 10 s and once for 5 min, and air-dried. Five microliters of Fluoromount ™ Aqueous Mounting Medium (Sigma-Merck) were applied to a microscope slide, and coverslips were applied and sealed.

Cells were examined using a Zeiss LSM 510 confocal microscope with a 64× lens objective and laser for fluorescence (DAPI, EX 330 to 380, Alexa 488, EX 460 to 500, and Alexa 647, EX 660 to 680). For better resolution, a 4× optical zoom was used. The percentage of piliated cells on each strain was quantified upon counting of RrgB positive cells related to the total DAPI positive cells. For cell counting, four different images from different sections were obtained on each experiment, and four independent experiments were performed for each strain. The results were statistically analyzed using Student’s *t*-test.

### 2.12. mRNA Half-Life Studies

Growing cultures of pneumococcal strains were diluted 1:50 in THY medium and incubated at 37 °C until OD_650_ ~ 0.3. At this point, 300 µg mL^−1^ Rifampicin was added and 1 mL samples were transferred to Eppendorf tubes containing 200 µL STOP solution (5% phenol equilibrated at pH 7 and 95% of ethanol) at time intervals, immediately immersed in liquid nitrogen and stored at −80 °C until needed. One milliliter of the sample corresponding to time 0 was extracted prior to Rifampicin addition. RNA was extracted using RNeasy Mini Kit and DNA was removed through on-column digestion with RNase-Free DNase Set (both from Qiagen, Madrid, Spain). RNA integrity was analyzed by gel electrophoresis and its concentration was determined using a Nanodrop 1000 machine (Nanodrop Technologies-ThermoFisher Scientific). Gene expression was determined by quantitative RT-PCR analysis as described in Section 2.5. Changes in transcript levels relative to time 0 on each strain were calculated using the 2^−ΔΔCt^ method. The mean of three independent experiments was plotted (semilogarithmically) as a function of time, and linear regression analysis (least-square) was used to identify the line that best fits the data. The decay rate constant was determined from the slope of the regression curve to obtain the half-lives.

## 3. Results

### 3.1. The Srn135 Coding Sequence Is Necessary for RrgA Translation 

The small, noncoding *srn135* was previously identified as an intergenic sRNA flanked by *rlrA* and *rrgA* Open Reading Frames (ORFs) [44]. However, previous work demonstrated that the *rrgA* transcript has a long 5′ untranslated region (5′-UTR) that starts in thymine (T) located 231 nucleotides (nt) upstream of the translation initiation codon [11]. The *srn135* coding sequence was mapped within this region. Therefore, instead of an intergenic RNA, *srn135* corresponds to the 5′-UTR of the *rrgA* transcript and is encoded by the *rrgA* gene. RrgA has been described as a negative regulator of RlrA and its inactivation is known to increase pili expression [35]. 

**Figure 2:**
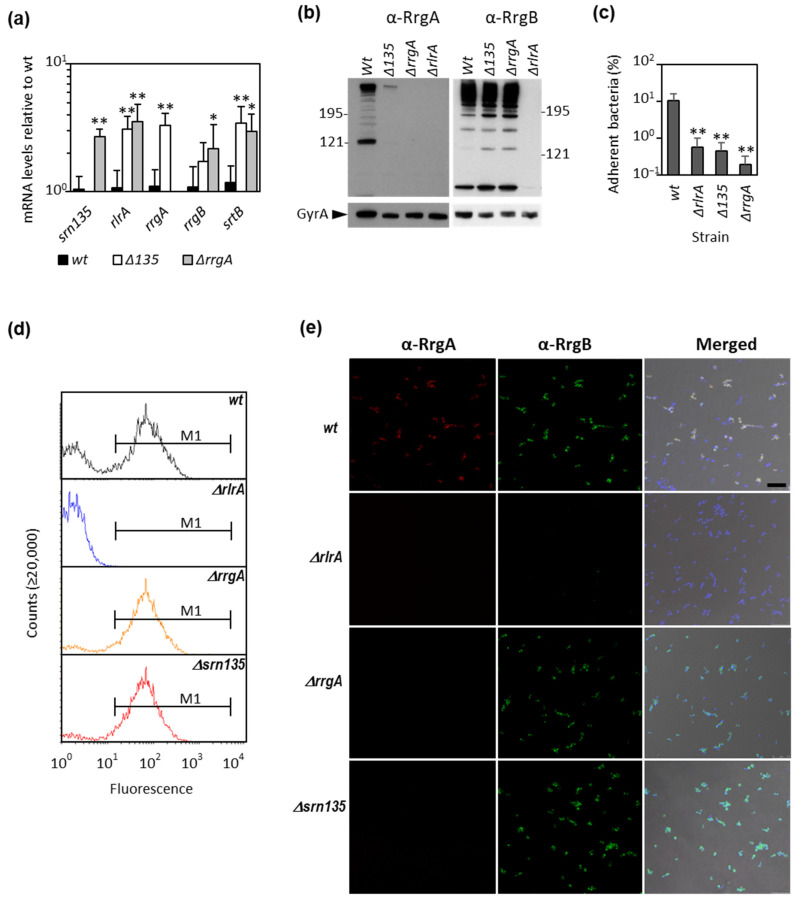
**Deletion of the *srn135* or *rrgA* gene results in an equivalent phenotype.** Pilus expression in parental (wt), Δ*135*, and Δ*rrgA* mutant strains were studied. (**a**) Cultures were grown to mid-exponential phase, and RNA was extracted and used in quantitative RT-PCR analysis. Data represent transcript levels relative to the parental strain using the ΔΔCT method. The results shown are the average of at least four independent experiments (±standard deviations). (**b**) Western blot of bacterial lysates using anti-RrgA (α-RrgA) or anti-RrgB (α-RrgB) as primary antibodies. A majority of the protein was found as a high molecular-weight ladder corresponding to the assembled pilus. Anti-GyrA antibody was used as a loading control. (**c**) Adherence to human epithelial lung cells was performed as described in Materials and Methods at an MOI of 50 bacteria per cell. Adherent bacteria were determined as the bound bacteria after 1 h incubation at 37 °C relative to the number of inoculated bacteria. Results are the mean of five independent experiments (±standard deviations). (**d**) For flow cytometry, bacteria were labeled with rabbit anti-RrgB as primary antibody and Alexa Fluor 488 goat anti-rabbit IgG as a secondary antibody. Sera of mice immunized with PBS were used as a negative control. A representative histogram of wt, Δ*rrgA*, Δ*rlrA*, and Δ*135* is shown. M1 denotes counted cells. (**e**) Bacteria were processed for immunofluorescence, stained with anti-RrgA (α-RrgA), anti-RrgB (α-RrgB) as primary antibodies, Alexa Fluor 488, and Alexa Fluor 647 as secondary antibodies, and DAPI for DNA staining. Imaging was performed with a Leica TCS-SP5-AOBS (Mannheim, Germany) confocal microscope. Merge signal of α-RrgA, α-RrgB, and DAPI staining is shown (Merged). The scale bar is 75 µm. Statistical significance was tested by Student’s *t*-test with significant data points being highlighted by asterisks (* *p* < 0.05; ** *p* < 0.01).

To explore whether *srn135* has a role in RrgA levels and, hence, in pilus expression, mutant strains lacking the coding region of *srn135* (Δ*srn135*), *rrgA* (Δ*rrgA*), or *rlrA* (Δ*rlrA*) were constructed. The deletion was performed using a self-deleting re-lox-ermAM cassette [56], which allowed deletion without introducing an antibiotic resistance cassette. Pili expression on the pneumococcal surface of the three mutant strains was first analyzed by transmission electron microscopy. As expected, the surface of Δ*rrgA* and wild-type strains were decorated by numerous long proteinaceous pili structures (Figure 1b,d) while Δ*rlrA* appeared as naked bacteria (Figure 1c). The Δ*srn135* strain exhibited a similar phenotype to that of Δ*rrgA* and the wild type, and long pili anchored to its surface were observed (Figure 1e). The effect of *srn135* inactivation on gene expression was assessed by qRT-PCR. Results in Figure 2a showed that Δ*srn135* had a similar phenotype to the Δ*rrgA* strain, with higher levels of *rlrA* (3.1-fold), *rrgB* (1.7-fold), and *srtB* (3.0-fold) than the wild-type strain. Inactivation of the *srn135* coding sequence also increased *rrgA* expression, showing 3.3-fold higher levels of transcript than the wild type. To assess whether such an increment correlated with protein levels, a Western blot analysis was performed using anti-RrgA and anti-RrgB antibodies (kindly provided by Prof. R. Malley from Children’s Hospital, Boston, USA). As expected, no RrgA or RrgB proteins were detected in the Δ*rlrA* mutant (Figure 2b), in agreement with the absence of transcription observed in this strain (data not shown). In the case of the Δ*srn135* strain, despite the higher levels of *rrgA* transcript, almost no RrgA protein was detected and, as occurs in Δ*rrgA*, RrgB levels were slightly increased compared to the wild type. The ability of the Δ*srn135* strain to adhere to A549 epithelial lung cells was also reduced to the levels of Δ*rlrA* and Δ*rrgA*, showing ≤ 0.5% of adherent cells compared to ~10% of the wild-type strain (Figure 2c). Therefore, deletion of *srn135* prevents translation, but not transcription, of the main pili adhesin of pneumococcal pilus, RrgA.

Pilus expression in pneumococcus is bistable, which means that within a clonal population containing the PI-1 islet, a subset of bacteria expresses pili on its surface, whereas, in another subset, pilus expression is either very low or virtually undetectable [35,37]. Slight differences in RlrA levels appear to function as an on/off switch controlling the percentage of piliated bacteria in a clonal population. We analyzed the bimodal expression of pili in exponentially growing cultures by flow cytometry using a primary polyclonal anti-RrgB antibody. The wild-type strain showed the expected bimodal pattern of expression with a 65.6 ± 0.4% of high-fluorescent and 35% of low-fluorescent bacteria (Figure 2d). Inactivation of *srn135* induced an increase in pili-expressing bacteria similar to that of the Δ*rrgA* strain, showing an 84.0 ± 4.1% and an 82.6 ± 4.1%, respectively (Figure 2d). To confirm these findings, we performed IIF and confocal microscopy using anti-RrgA and anti-RrgB primary antibodies (Figure 2e). As occurred with Δ*rrgA*, the Δ*srn135* strain was positive for RrgB but not for RrgA, while the parental strain was positive for both. Moreover, the counting of fluorescent bacteria rendered similar results to flow cytometry, showing 95.7 ± 2.6% and 90.6 ± 6.1% of piliated bacteria in Δ*rrgA* and Δ*srn135* strains, respectively, compared to a 73.3 ± 2.7% in the wild-type strain. These findings indicated that the *srn135* coding sequence at the 5′-UTR of the *rrgA* transcript is required for RrgA translation and that its deletion is sufficient to cause the same effect on pilus expression as the deletion of the whole *rrgA* ORF.

### 3.2. The Srn135 sRNA Is Generated upon Processing of RrgA Transcript

**Figure 3:**
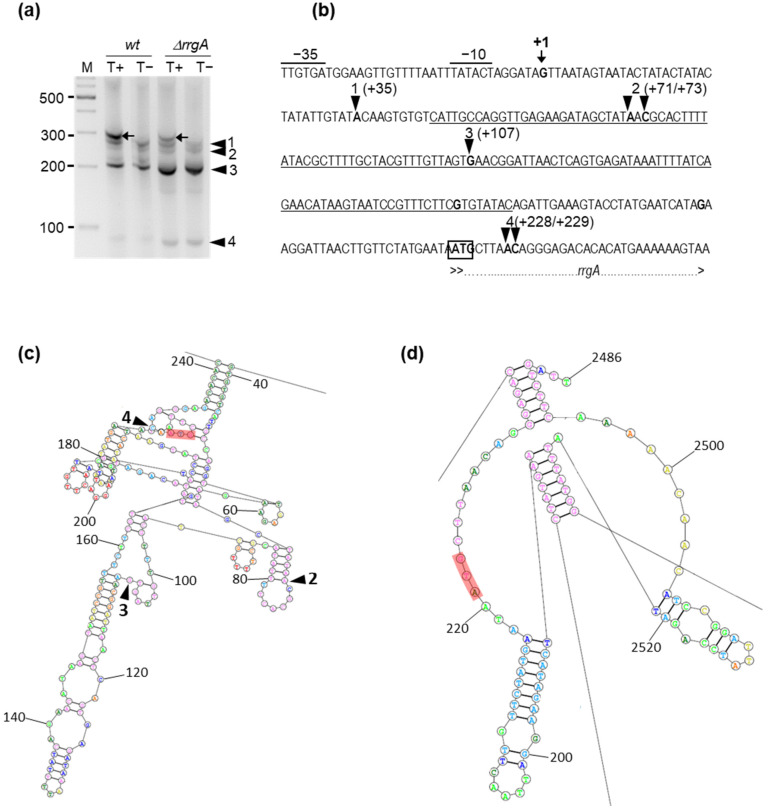
***srn135* is generated upon processing of the *rrgA* transcript.** (**a**) 5′ RACE mapping of the *srn135* small RNA and the *rrgA* transcript. Reverse transcription was carried out on 7 µg of total RNA extracted from the wild type andΔ*rrgA* mutant strains as described in Materials and Methods. PCR signals upon treatment with TAP (T+) or without treatment (T-) were separated in a 4% agarose gel. The RACE product that was present only in TAP-treated samples (black arrow) corresponded to the 5´-end of the full-length transcript, whereas RACE products 1, 2, 3, and 4 (arrowheads) present in TAP-treated and non-treated samples are likely to be processing fragments. (**b**) Sequence of the region comprising the 5′ UTR of *rrgA* mRNA and the beginning of the ORF. Transcriptional start site (highlighted with a black arrow) was identified as a G located 7 nt downstream of the −10 box. The nucleotides corresponding to the 5′-end of RACE products 1, 2, 3, and 4 (arrowheads), are highlighted in bold. The ATG of *rrgA* is delimited by an empty box and the beginning of the ORF is indicated as a dashed line. The putative −35 and −10 of the promoter are indicated and the nucleotide sequence of *srn135* is underlined. Secondary structure prediction of the *rrgA* transcript before (**c**) and after processing at site 3 (**d**) was performed with RNAstructure web server tools. A diagram showing the predicted lowest free energy secondary structures obtained for the 5′-end of full-length (left panel) and the processed *rrgA* mRNA (right panel). Numbering corresponds to nucleotide position from the +1 site. Main cleavage sites are labeled with arrowheads and the ATG codon of *rrgA* is labeled in red.

The *srn135* sRNA accumulates as an independent 126 nt RNA in TIGR4 cells, as demonstrated by Northern blot [44]. To explore how *srn135* is generated, we mapped the 5′-end of the transcript by 5′ RACE [61]. This method allowed the distinguishing between the 5′-ends of primary transcripts from those generated by processing. A unique 5′ RACE product was detected only in TAP-treated samples (carrying the 5′-triphosphate group characteristic of primary transcripts) indicative of only one transcription start site (TSS) for both *srn135* and *rrgA* (band 1 in Figure 3a). Sequencing of this RACE product showed that this TSS corresponded to a G located two nucleotides downstream of the +1 site previously described by primer extension [11]. Given the discrepancy with previous results, three independent experiments were performed, and the same result was obtained. Consensus −10 and −35 sequences were found 7 nt upstream the G identified as +1 site (Figure 3b), which supported our results.

The gel in Figure 3a also showed four 5′ RACE products present in both TAP-treated and non-treated samples, likely derived from processed molecules. Their corresponding bands were gel extracted, cloned, and sequenced allowing us to infer their respective 5′-end. The most abundant product corresponded to band 3 and its 5′-end mapped in the +107 nt within *srn135*. Cleavage at this site would inactivate *srn135* and generate a processed *rrgA* transcript with a substantially shorter 5′-end. To elucidate its consequences in RNA folding, we conducted a secondary structure prediction of the full-length and processing-derived fragment of *rrgA* mRNA using RNAstructure web server tools [62]. The 5′-UTR of the full-length transcript was predicted to be highly structured between nucleotides +39 and +241, which include the ATG codon (Figure 3c). However, cleavage at nucleotide +107 appeared to disrupt the structure. The predicted 5′-end of the processed fragment was mainly unfolded and the ATG codon was exposed (Figure 3d). Although weaker, bands 1, 2, and 4 were obtained in all experiments, and their respective 5′-ends were located in different sites along the 5′-UTR of *rrgA* (Figure 3b). Interestingly, the 5′-end of product 4 was mapped in nucleotide +228, 5 nt downstream of the *rrgA* start codon. Cleavage at this site would inactivate *rrgA* and generate a functional independent *srn135*. Therefore, the 5′-UTR of the *rrgA* transcript seems to undergo different processing events, some of which may result in the accumulation of *srn135* as an independent RNA.

### 3.3. Overexpression of Srn135 in Trans Increases Pilus Expression

**Figure 4:**
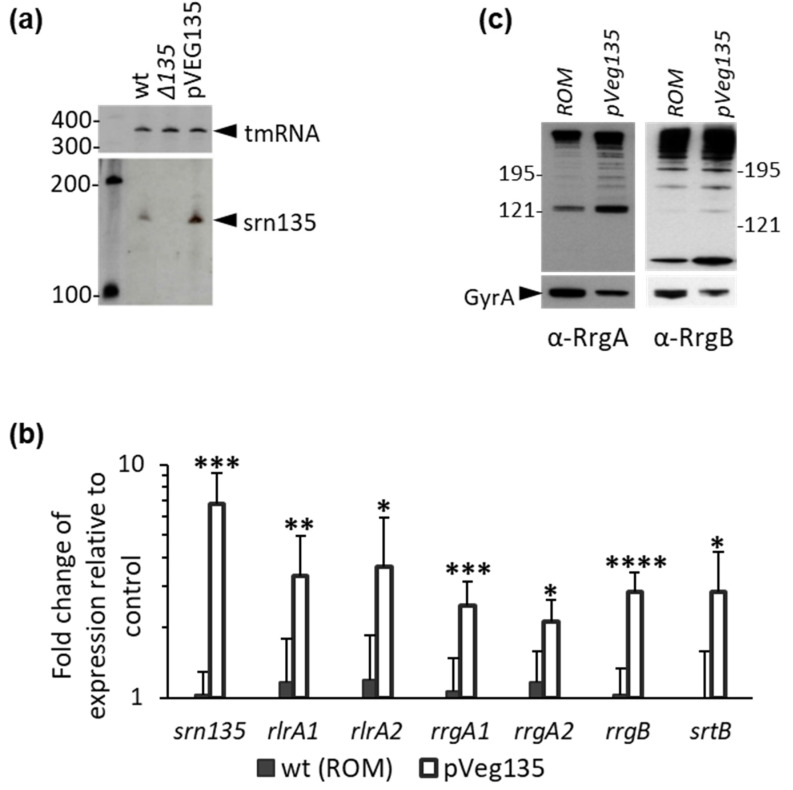
**Overexpression of *srn135* increases the expression of pili genes.** Pilus expression in TIGR4(pveg135) overexpressing *srn135* (*pveg135*) was studied and compared to the TIGR4(pROM) harboring the empty plasmid (*ROM*) as a control strain. (**a**) Northern blot showing *srn135* expression on the parental (wt), the Δ*135* mutant, and the overproducer strain *(pveg135*). A band corresponding to the independent *srn135* was accumulated in wt and *pveg135*, showing the latter higher levels of accumulation. The tmRNA was used as a loading control. Specific bands are indicated by arrowheads. The length of RNA in nucleotides is indicated on the left. (**b**) Expression of *srn135*, *rlrA*, *rrgA*, and *rrgB* was analyzed by qRT-PCR in TIGR4(pROM) and TIGR4(pveg135). For *rlrA* and *rrgA* two primer pairs were used, one matching the 5′ end (rlrA1 and rrgA1) and the other matching the 3′-end of the transcript (rlrA2 and rrgA2). Data represent transcript levels relative to the control strain using the ΔΔCT method. The results shown are the average of at least four independent experiments. (**c**) Western blot of bacterial lysates using anti-RrgA (α-RrgA) or anti-RrgB (α-RrgB). A majority of the protein was found as a high-molecular-weight ladder corresponding to the assembled pilus. Antisera against GyrA was used as a loading control. Protein molecular weights are indicated on the left. Statistical significance was analyzed by a Student’s *t*-test with significant data points being highlighted by asterisks (* *p* < 0.05; ** *p* < 0.01; *** *p* < 0.001; **** *p* < 0.0001).

To investigate whether *srn135* has a regulatory function, we cloned its coding sequence in a pROM plasmid [51] under the control of the constitutive vegT promoter (P*_vegT_*) [49]. The resulting pveg135 plasmid was introduced in wild-type TIGR4 cells (wt(pveg135)) and accumulation of a band corresponding to *srn135* was confirmed by Northern blot analysis (Figure 4a). The wt(pveg135) strain exhibited ~6.8 higher levels of *srn135* than the wild-type pneumococci harboring the pROM vector (wt(pROM)) as measured by qRT-PCR (Figure 4b). The transcript level of the other pili operon genes was also analyzed in both the overproducer and the control strain. To ensure that the measurement corresponded to full-length *rlrA* and *rrgA* transcripts, two primer pairs located at the 5′- (primer pair 1) or the 3′-end (primer pair 3) were used. As shown in Figure 4b, overexpression of *srn135* resulted in ~3.5-fold higher levels of *rlrA* full-length transcript than the control strain (3.3-fold with rlrA1 and 3.7-fold with rlrA2 primer pair). In the case of *rrgA*, 2.5- and 2.1-fold increments were observed with rrgA1 and rrgA3 primer pairs, respectively, indicating a ~2.3-fold accumulation of the full-length transcript. Levels of the other pili genes *rrgB* and *srtB* were also significantly higher in wt(pveg135) than in the control strain. Accordingly, RrgA and RrgB protein levels were 4.9 ± 0.8- and 2.2 ± 0.05-fold higher in wt(pveg135) than in wt(pROM), respectively, as measured by the Western blot (Figure 4c). However, no significant increase in adherence was observed in the *srn135* overproducer strain compared to control bacteria (not shown). These results suggest that the accumulation of *srn135* in *trans* positively regulates the levels of *rlrA* messenger, resulting in higher expression of the other pili genes and higher amounts of protein.

**Figure 5:**
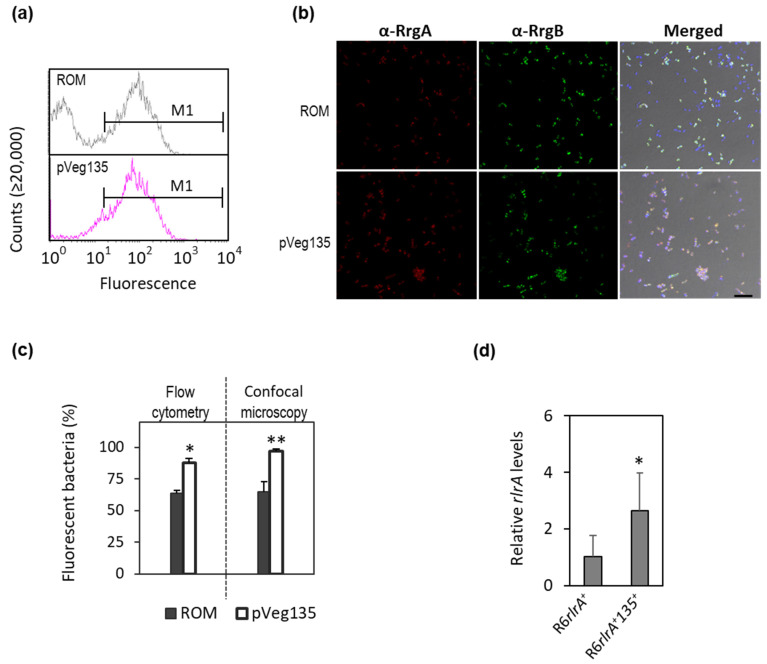
**Overexpression of *srn135* causes a monophasic pilus expression pattern.** The percentage of piliated bacteria in TIGR4(pveg135) (*pveg135*) and the TIGR4(pROM) control strain (*ROM*) was studied. (**a**) Flow cytometry was used to assess the pilus expression pattern. Bacteria were labeled with anti-RrgB as primary antibody and Alexa Fluor 488 goat as a secondary antibody. Sera of mice immunized with PBS were used as the negative control. A representative histogram of TIGR4(pROM) and TIGR4(pveg135) is shown. M1 denotes counted cells. (**b**) For immunofluorescence, samples were processed as described in Materials and Methods and stained with anti-RrgA (α-RrgA) and anti-RrgB (α-RrgB). Imaging was performed with a Leica TCS-SP5-AOBS (Mannheim, Germany) confocal microscope. The right panel represents the merged signal of α-RrgA (red), α-RrgB (green), and DAPI staining (blue). The scale bar is 75 µm. (**c**) The percentage of piliated bacteria on each strain was determined by flow cytometry (left) and confocal microscopy (right). Data from flow cytometry are the mean of three independent experiments. Data from confocal microscopy are the mean of four independent experiments (*n* = 3 fields on each). (**d**) The effect of *srn135* on *rlrA* expression was studied in a non-piliated R6 pneumococcal strain harboring an exogenous *rlrA* gene inserted in the *bgA* locus (*R6rlrA*). Transcript levels were determined by qRT-PCR in the *R6rlrA* containing the pveg135 plasmid (*R6rlrA + 135*) or the empty pROM vector (*R6rlrA*). Data represent transcript levels relative to the control *R6rlrA* strain using the ΔΔCT method. The results shown are the average of four independent experiments. Statistical significance was analyzed by Student’s *t*-test with significant data points being highlighted by asterisks (* *p* < 0.05; ** *p* < 0.01).

The percentage of piliated bacteria in wt(pveg135) was also significantly higher than in the wt(pROM). Flow cytometry analysis revealed only one peak of highly fluorescent bacteria in the wt(pveg135) strain (87.6 ± 3.6% of the whole population), while a typical bimodal pattern was observed in the control strain (with 63.4 ± 2.3% of high-fluorescent bacteria) (Figure 5a,c). Similarly, IIF and confocal microscopy demonstrated that almost the whole wt(pveg135) population was positive for RrgA and RrgB, with a 96.9 ± 2.0% of piliated bacteria, compared to 64.9 ± 7.6% showed by the wt(pROM) (Figure 5b,c).

The high pilus expression phenotype within a clonal population is very stable, and it has been postulated that this phenomenon may lead to the misidentification of pilus expression regulators [34]. To circumvent this issue, we cloned the *rlrA* encoding gene in the *bgaA* chromosomal locus of the R6 non-piliated pneumococcal strain (R6*rlrA*), and the pveg135 plasmid overexpressing *srn135* or pROM vector was introduced by transformation. Expression analysis of *rlrA* in both strains by qRT-PCR showed that the *srn135*-containing strain (R6*rlrA*(pveg135)) exhibited 2.7-fold higher levels of *rlrA* transcript compared to the non-*srn135*-containing strain (R6*rlrA*(pROM)) (Figure 5d). The differences observed between the two strains were not influenced by the bimodal expression of pilus and are exclusively due to the presence of *srn135*, confirming the positive regulation of *srn135* on the *rlrA* levels.

### 3.4. Srn135 Modulates RlrA Levels by Affecting Its Decay

**Figure 6:**
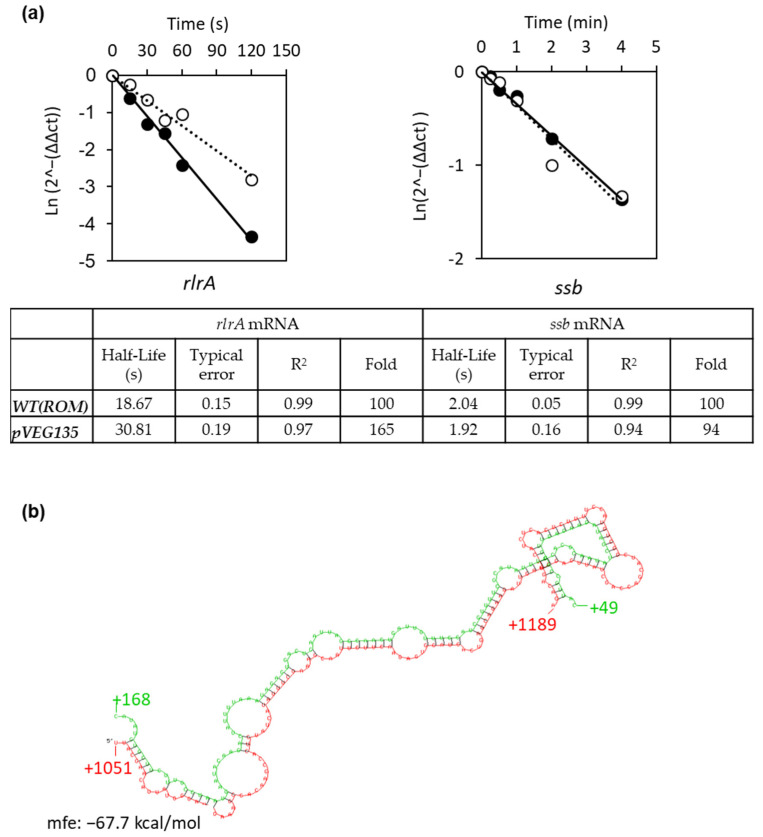
**Overexpression of *srn135* increases the stability of the *rlrA* mRNA.** (**a**) Half-lives of *rlrA* (left panel) and *ssb* (right panel) mRNAs were measured in TIGR4(pROM) (black circle) and TIGR4(pveg135) (white circle) strains by quantitative RT-PCR. Bacterial samples were taken after the addition of rifampicin at the indicated times and RNA was extracted as described in Materials and Methods. Data are presented as the transcript levels relative to time 0 on each strain using the ΔΔCT method over time. Half-lives were calculated from the slope of the regression curve. Experiments were performed in triplicate. Typical errors and R^2^ of the regression line are shown. Half-lives of mRNAs in TIGR4(pROM) were arbitrarily reported as 100 and the values of TIGR4(pveg135) were relative. (**b**) Potential binding of *rlrA* mRNA (red) and *srn135* (green) was analyzed with the RNA hybrid program using the web tool on the Bielefeld Bioinformatics Server (https://bibiserv.cebitec.uni-bielefeld.de/rnahybrid accessed on 27 July 2021). Graphical output of the energetically most favorable hybrid between both RNAs, with a minimum free energy (MFE) of −67.7 kcal/mol shown.

Our results demonstrated that *rlrA* levels directly correlate with *srn135* levels. This might be due to the increased stability of the *rlrA* mRNA mediated by *srn135*. All efforts to detect the *rlrA* mRNA in TIGR4 cells by the Northern blot were unsuccessful, suggesting very low levels for this transcript. Therefore, we tested the effect of *srn135* on the stability of *rlrA* mRNA by qRT-PCR in both the wt(pveg135) and wt(pROM). Levels of the transcript were measured at different time points after the addition of rifampicin to the culture. The results demonstrated that the half-life of the *rlrA* mRNA was only 18.7 seconds (s), which is likely the reason why the mRNA could not be detected by the Northern blot (Figure 6a). Overexpression of *srn135* in the wt(pveg135) increased the *rlrA* half-life to 30.8 s, meaning that the *rlrA* transcript was 165% more stable upon overproduction of *srn135* (with a typical error of 0.15 and 0.19, and an R^2^ value of 0.98 and 0.97 for the regression curves of wt(pROM) and wt(pVeg135), respectively). By contrast, the stability of the *ssb* transcript was not affected by *srn135* and the half-life was about 2 min in both strains, indicating that the *srn135* effect observed on the *rlrA* stability was specific.

## 4. Discussion

In this report, we studied the role of *srn135* as a novel regulator of PI-1 expression. This 126 nt RNA was first identified in a TIGR4 strain as an intergenic RNA located between *rlrA* and *rrgA* loci [44], and its independent expression was demonstrated. Here, we provided evidence that *srn135* corresponds to the 5′-UTR of the *rrgA* transcript. Our findings demonstrated that the deletion of *srn135* increased *rrgA* transcription but drastically diminished translation. Accordingly, the Δ*srn135* mutant exhibited an equivalent phenotype to that of Δ*rrgA*, increasing pili expression and impairing adherence. Mapping the 5′-end of the *rrgA* transcript revealed only one transcriptional start site for both *srn135* and *rrgA*. In addition, several processing sites were identified along the 5′-UTR. A major processing fragment generated by cleavage at position +107 (band 3 in Figure 3a,b) would inactivate *srn135*, while cleavage at positions +228 and +229 (band 4 in Figure 3a,b) would result in its accumulation as an independent RNA disrupting *rrgA*. Cleavage at +107 appears to affect folding drastically, turning a highly structured 5′-end into another eminently unfolded. This cleavage is likely to have important consequences for RrgA translation since no Shine–Dalgarno (SD) sequence can be identified in the *rrgA* gene. The ATG codon of *rrgA* mRNA is predicted to be included in a secondary structure barely accessible to ribosomes (Figure 3c), and only after cleavage would it be available for translation (Figure 3d). Therefore, cleavage of the *rrgA* transcript at nucleotide +107 appears to be a necessary step for proper translation. In fact, genome-wide studies in various groups of prokaryotes revealed that the absence of secondary structures in the initiation translation region is necessary to allow translation of mRNAs lacking SD, and this local unfolding is sufficient to initiate SD-independent translation [63].

Secondly, our findings demonstrated that increasing the levels of *srn135* RNA had a positive effect on pilus expression, turning a biphasic expression into monophasic with almost 100% of the population expressing pili. Such an effect was associated with a higher stability of the *rlrA* mRNA. A 6.8-fold increase in *srn135* levels in the overproducer strain induced a 64% increment in the *rlrA* half-life. Several studies attributed a direct role to sRNAs in the modulation of mRNA stability [64,65,66]. This often requires a direct interaction between both RNAs, inducing conformational changes that alter its susceptibility to ribonucleases (RNases). Therefore, stabilization of *rlrA* mRNA might occur via duplex formation. A search for potential duplexes between both RNAs using the RNAhybrid server [67] rendered a best hit with minimum free energy (MFE) of -67.7 kcal/mol (Figure 6b). In the predicted duplex, *srn135* hybridizes within the coding region of the *rlrA* transcript between nucleotides +1051 and +1189 (Figure 6b). Such interaction is likely to induce conformational changes in the *rlrA* transcript, which could either generate protective secondary structures or destabilize stem-loops necessary for cleavage, thereby affecting mRNA fate. Similar mechanisms of regulation were demonstrated in *Salmonella* and *Escherichia coli* where the sRNAs *MicC* and *RyhB* increased degradation of some mRNA targets through base-pairing within the coding region or the intergenic region [68,69,70]. Other sRNAs increase mRNA stability, such as the VR–RNA, which induces stability of collagenase in *Clostridium perfringens* [71], or FasX in the groups A *Streptococcus*, which binds to the 5′-end of streptokinase mRNA and promotes stability [72]. Therefore, sRNA-mediated regulation of mRNA stability is a common mechanism to modulate gene expression in bacteria.

In summary, our data are consistent with an opposite action of *srn135* as a positive regulator and RrgA as a negative regulator of pilus expression. This highlights the potential role of alternate RNA cleavage mediated by RNases in the balance between them. Differential cleavage of the 5′-UTR of *rrgA* could be the result of a concert action between endo- and exo-ribonucleases, thus generating either a functional *rrgA* or an independent *srn135*. Knowledge of pneumococcal RNases is scarce, but it has been recently demonstrated that the endoribonuclease RNase Y and the exoribonuclease PNPase work in concert to regulate the processing and decay of several regulatory RNAs, in particular, those characterized by the presence of 5′-cis-acting regulatory elements [73]. Likewise, RNase Y has been postulated to play important roles in the processing and maturation of sRNAs, which are generated upon cleavage of larger transcripts [73]. These RNases are thought to have a global impact on virulence through sRNA regulation, and the modulation of pilus expression could be another example of this function.

The ability to regulate the assortment and abundance of virulence factors is critical for bacterial pathogens to undergo a productive infection and the pilus-1 type provides an important selective advantage during pneumococcal colonization and pathogenesis. The relevance of RrgA during *S. pneumoniae* colonization and invasion has been well documented (reviewed in [74]) and the processing of its 5′-UTR may play a role in the transition from colonization to dissemination during infection. Furthermore, our results provide new insight into the sRNA-mediated regulatory mechanisms in the pneumococcus. Almost 200 sRNAs have been identified in *S. pneumoniae* so far; however, our knowledge about their biological function is still in its beginning. In this report we demonstrated that *srn135* is a new post-transcriptional regulator that modulates mRNA stability, evidencing a new mechanism of RNA regulation in the pneumococcus.

## Figures and Tables

**Table 1:** Strains and plasmids used in this study.

Bacterial Strain	Description	Source
TIGR4	Serotype 4 clinical isolate	[52]
TIGR4Δ*135*	TIGR4 *srn135::lox72*	This study
TIGR4Δ*rlrA*	TIGR4 *rlrA::lox72*	This study
TIGR4Δ *rrgA*	TIGR4 *rrgA::lox72*	This study
TIGR4(ROM)	TIGR4 [pROM]	This study
TIGR4(pveg135)	TIGR4 [pROM-P*_vegT_*-135]	This study
R6	Non-encapsulated strain derived from the capsular type 2 clinical isolate strain D39	Laboratory collection
R6*rlrA*	R6 *bgaA::pTP2-rlrA*	This study
R6*rlrA*(ROM)	R6 *bgaA::pTP2-rlrA*[pROM]	This study
R6*rlrA*(pveg135)	R6 *bgaA::pTP2-rlrA*[pveg135]	This study
**Plasmids**		
pTP2	Integrative plasmid catalyzing integration in the loci *spr0564* and *bgaA* of the R6 genome	[49]
pTP3	Integrative plasmid derived from pTP2	[49]
pROM	pLS1ROM lacking PM promoter	[51]
pTP3-*135*	pTP3 containing a 197 bp fragment of the *rrgA* 5′-UTR	This study
pROM-P*_vegT_*-135	pROM containing the *srn135* coding sequence under the control of P*_vegT_* promoter derived from pTP3-135	This study
pTP2-*rlrA*	pTP2 containing *rlrA* coding sequence	This study

**Table 2:** List of primers used in this study. The underlined sequence indicates enzyme-restriction sites. RT: reverse transcription reaction.

Primer	Nucleotide Sequence 5′ to 3′	Application
135-F	TTGCCAGGTTGAGAAGATAGC	RT/Northern blot
135-R	CACGAAGAAACGGATTACTTATGTT	RT/Northern blot
rlrA-F1	TGGAAGTATGGATTGGGTCA	RT/Northern blot
rlrA-R1	TGTTGGCATGTGGCTCTAAG	RT/Northern blot
rlrA-F2	GACAAAGTTGCCTCTGTTACA	RT
rlrA-R2	CTGATAGATGAGACGCTGT	RT
rrgA-F1	CCGCTGGATGTCGTTATCTT	RT
rrgA-R1	CATTCAATCGCTTTCCGTTT	RT
rrgA-F2	CAATGGTCGAACAACCTTAC	RT
rrgA-R2	GTCAACTTACCATCTTCACCT	RT
rrgB-F	ATTGCCGGTGTTATGTTCGT	RT
rrgB-R	GCTGGTAAATTTGCCGTGTT	RT
srtB-F	AAAAGCAACGTTGGATGAGG	RT
srtB-R	GTCAATAACGGGGATTTCCA	RT
16s-F	AGCGTTGTCCGGATTTATTG	RT
16S-R	CATTTCACCGCTACACATGG	RT
Primer9	GGGGACGCGTTGGCTTACCGTTCGTATAG	KO construction
Primer10	GGGGCCATGGTCGATACCGTTCGTATAATGT	KO construction/5′-RACE
135KO-Mlu	CGCGACGCGTATTACTATTAACTATCCTAGTATAAATTAAA	KO construction
13KO-Nco	CGCGCCATGGTACCTATGAATCATAGAAGGAT	KO construction
rlrAKO-Mlu	CGCGACGCGTCTCATCTATCAGACAA	KO construction/5′-RACE
rlrAKO-Nco	CGCGCCATGGAATTTCCGACTTTATT	KO construction
rrgAKO-Mlu	CGCGACGCGTGCCTTCTGAAATATCTTTC	KO construction/5′-RACE
rrgAKO-Nco	CGCGCCATGGGAGGAGTTCTATTATACAC	KO construction
rlrAup-F	ACGTCTGTTATCAAGAATGGTCA	KO construction
rrgAdown-R	AACTTGGTGGTTCACAGGTGTAT	KO construction
135up-F	TTTGGACTCAGGGAACTCAAGT	KO construction
135down-R	TTGGTATTGGTTGTAAAACTCGTCT	KO construction
135F-blunt	GTTAATAGTAATACTATACTATACTATATTGTATACAAGT	Cloning of *srn135*
135R-Bam	CGCGGATCCTTCTATGATTCATAGGTACTTTC	Cloning of *srn135*
PvegF-Hind	CGCAAGCTTTGCATGCTTGGACTCC	Cloning of *srn135*
rrgA-RT	GACTGGTTTCAGGCGTTTCT	5′-RACE
RNA adaptor	GAUAUGCGCGAAUUCCUGUAGAACGAACACUAGAAGAAA	5′-RACE
RACE-5′	GATATGCGCGAATTCCTGTAG	5′-RACE
RACE-5′-inner	AATTCCTGTAGAACGAACACTAGAA	5′-RACE
RT-RACE	ACAGCACAGTCCTGCAACTG	5′-RACE
wt-inner	ACAGTCCTGCAACTGCCTTC	5′-RACE
rlrA-Sph	GCGCGGCATGCGATATTTTTATCACATATTTTTTTATAGAACGAC	R6*rlrA* construction
rlrA-Bam	CGCGGATCCTTATGGGACTTTTTTGATACTC	R6*rlrA* construction
SP6	ATTTAGGTGACACTATAG	Sequencing
T7	TAATACGACTCACTATAGGG	Sequencing
